# phylotypr: an R package for classifying DNA sequences

**DOI:** 10.1128/mra.01144-24

**Published:** 2025-01-14

**Authors:** Patrick D. Schloss

**Affiliations:** 1 Department of Microbiology & Immunology, University of Michigan, Ann Arbor, Michigan, USA; Loyola University Chicago, Chicago, Illinois, USA

**Keywords:** 16S rRNA, microbial ecology, microbiome, bioinformatics, classification

## Abstract

The phylotypr R package implements the popular naive Bayesian classification algorithm that is frequently used to classify 16S rRNA and other gene sequences to taxonomic lineages. A companion data package, phylotyprrefdata, also provides numerous versions of taxonomic databases from the Ribosomal Database Project, SILVA, and greengenes.

## ANNOUNCEMENT

Since it was published in 2007, the naive Bayesian classifier has been the most popular and performant tool for classifying 16S rRNA gene sequences (1). The method calculates the probability distributions of k-mers (typically 8-mers) across a reference collection and within each genus represented in the collection. These probabilities are used within a pseudo-bootstrapping procedure to classify unknown sequences and assign a confidence score to that classification. The confidence scores are used to prune the Linnaean taxonomy to the deepest possible taxonomic level with sufficient confidence (typically 80%). The algorithm was been made available by the original developers as an application coded in Java; a wrapper for the original code was available in QIIME (2). A C++ version has been available in mothur, and a Python version in QIIME2 (3, 4). Until March 2023, users could classify sequences with an online interface at the Ribosomal Database Project (RDP); this interface is no longer available. The RDP developers continue to update their code and the database through their GitHub and Sourceforge-based repositories (5).

Considering the growing popularity of the R programming language among microbial ecologists (6–10), I developed an R-based version of the algorithm that is available as the phylotypr package. Users can install phylotypr via CRAN or through the devtools package’s install_github function. Classification consists of two steps. First, the reference sequences and taxonomies are used to calculate kmer-based probabilities with the build_kmer_database function. Users can specify their desired kmer size when generating the database. These probabilities can be recalculated for each R session or saved as an R data file. Their calculation can be completed within several seconds. Second, user-supplied sequences can be classified using the reference database with the classify_sequence function. Accessory filter_taxonomy and print_taxonomy functions allow the user to output the results of their classifications using a minimum confidence score threshold. A detailed vignette is available within the phylotypr package that demonstrates how to install the package, use its functions, and parallelize its performance using the furrr package. The R-based execution time is comparable with or faster than that found when using the classify.seqs mothur function written in C++.

Many microbial ecologists have benefited from training the algorithm using reference sequences and taxonomies curated by the RDP as well as other providers including greengenes and SILVA (5, 11–16). For demonstration purposes, phylotypr includes a small reference database using version 9 of the RDP’s reference. A companion data package, phylotyprrefdata, is available on GitHub and can be installed using the install_github function from the devtools package. The current version of the data package (v0.1.0) includes all publicly available versions of the references from each of the RDP, greengenes, and SILVA references. Because of the size of the package (150 MB), it is too large to post to CRAN. I plan to make regular updates to the data package as new versions of databases become available. Users can also provide their own reference data to classify genes other than the 16S or 18S rRNA gene to improve the classification of lineages that are poorly represented in the references.

## Data Availability

phylotypr is available through CRAN and developmental versions are available through the project’s GitHub website (https://github.com/mothur/phylotypr). A pkgdown version of the documentation is hosted at (https://mothur.org/phylotypr). The phylotyprref data package is available through the project’s GitHub website (https://github.com/mothur/phylotyprrefdata). The phylotypr package is available under the GPL (v3) license and the phylotyprrefdata package is available under the MIT open source license.
